# Supplementary material for: FGF9-Associated Multiple Synostoses Syndrome Type 3 in a Multigenerational Family
Source: Genes (Basel). 2023 Mar 15;14(3):724. doi: 10.3390/genes14030724 (PMC10048304; doi:10.3390/genes14030724)
Supplement: Supplementary file 1 [file genes-14-00724-s001.zip › genes-2229953-supplementary.pdf]

GCAT TACG GCAT genes

Supplementary Materials

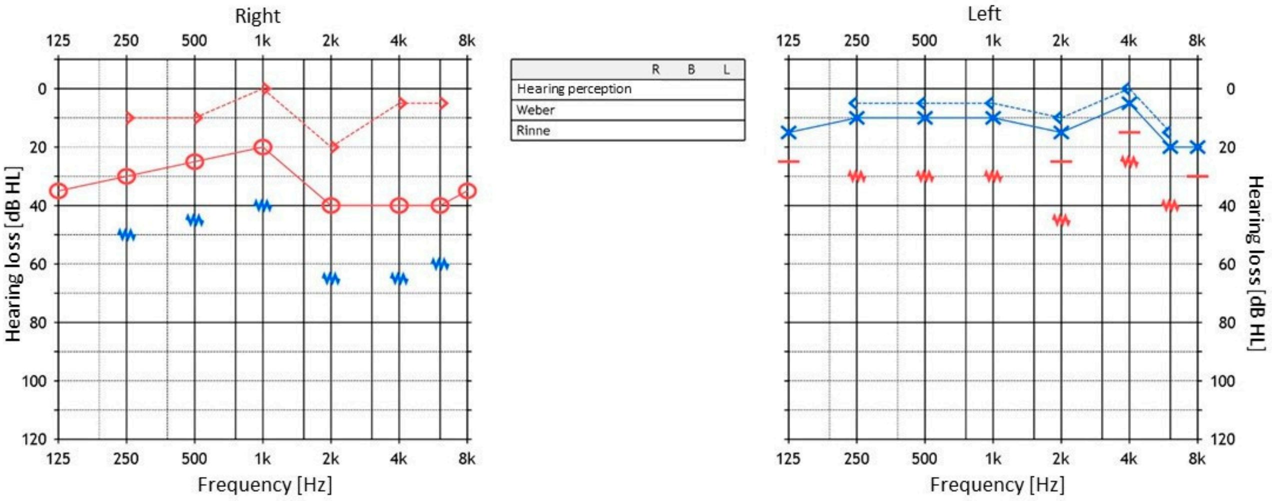

Figure S1: Audiometric evaluation of patient VI.2.: normal hearing on the left side. On the right side a mild conductive hearing loss of 20 dB in the low and middle frequencies and 30 dB in the higher frequencies was measured. The bone conduction threshold showed normal inner ear with hearing levels at 0-10 dB except for a slightly increased bone conduction threshold at 2 kHz to 20 dB, which reminds a Carhart-notch indicative of otoscleroses.

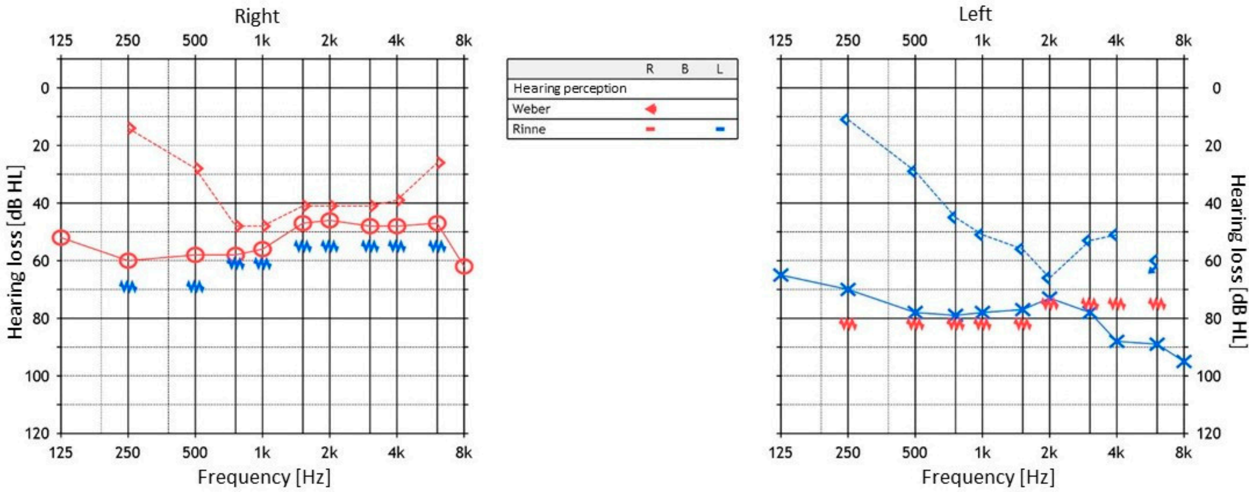

Figure S2: Audiometric evaluation of patient V.1.: On the right side an air conduction threshold of 50-60 dB HL was measured. There was an air-bone-gap of 30-40 dB at 250 Hz and 500 Hz. On the left side there was an air conduction threshold of 70-90 dB HL with an air-bone-gap of 40-50 dB in the lower frequencies, 30 dB in the middle frequencies, 5 dB at 2 kHz and 30-40 dB from 2-4 kHz.
